# Protected Metallic Edge States in a Graphene-Capped Quantum Spin Hall System

**DOI:** 10.1021/acsnano.6c14197

**Published:** 2026-09-30

**Authors:** Huu Thoai Ngo, Zamin Mamiyev, Niclas Tilgner, Andres David Peña Unigarro, Sibylle Gemming, Thomas Seyller, Christoph Tegenkamp

**Affiliations:** † Institut für Physik, 38869 Technische Universität Chemnitz, Reichenhainer Str. 70, Chemnitz D-09126, Germany; ‡ Research Center for Materials, Architectures and Integration of Nanomembranes (MAIN), Chemnitz 09126, Germany; § TEM-Center, Chemnitz University of Technology, Erfenschlager Straße 73, Chemnitz D-09125, Germany

**Keywords:** quantum spin Hall system, bismuthene, edge states, epitaxial graphene, proximity coupling

## Abstract

Epitaxial bismuthene on SiC(0001) hosts symmetry-protected metallic edge states within a large bulk band gap, establishing it as a promising two-dimensional topological insulator for high-temperature quantum spin Hall (QSH) transport. Here we realize bismuthene islands by intercalating Bi beneath zero-layer graphene on SiC(0001) followed by hydrogen treatment, yielding well-defined edge terminations. Spectroscopic measurements demonstrate the metallic edge states originating from the underlying bismuthene layer. The graphene overlayer interacts only weakly with the bismuthene, preserving its topological character while providing environmental protection. Overall, our results establish graphene-capped bismuthene as a robust and tunable platform for studying correlated quantum states, with the one-dimensional edge channels exhibiting signatures of electronic correlation effects.

## Graphical abstract

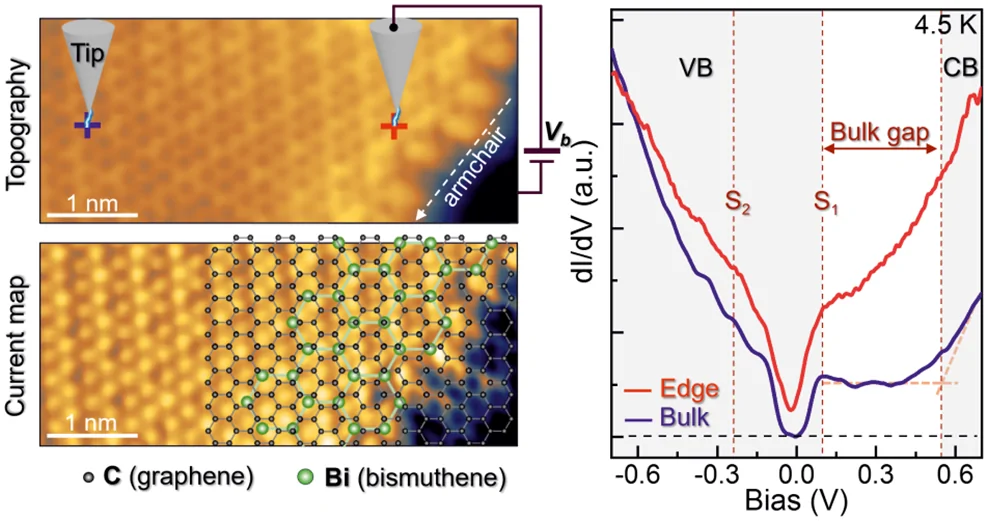

## Introduction

Two-dimensional (2D) materials provide a versatile platform for emergent quantum phenomena, including dimensionality-dependent electronic structure^1,2^ symmetry breaking^3,4^ and non-trivial topology,^5^ absent in bulk systems. Among them, 2D topological insulators (TIs) host symmetry-protected helical metallic edge states within a bulk band gap, giving rise to the quantum spin Hall (QSH) effect.^6,7^ These edge channels enable dissipationless transport^8^ and are robust against nonmagnetic disorder, offering potential applications in the fields of spintronics and topological quantum computing.^9–11^

The quantum spin Hall (QSH) effect was first proposed by Kane and Mele in graphene with spin–orbit coupling.^6^ Since then, it has been realized in diverse two-dimensional topological insulators, including HgTe/CdTe quantum wells (QWs),^12^ InAs/GaSb QWs,^13^ InAs/GaInSb/InAs QWs,^14^ group-IV monolayers (silicene,^15^ germanene,^16^ stanene,^17^ transition-metal dichalcogenides (1T′-WTe_2_,^18^ 1T′-MoS_2_),^19^ and even layered compounds such as Bi_4_Br_2_I_2_ ^20^ and ZrTe_5_.^21^

Compared to these, bismuthene is a monoelemental QSH system with a large band gap that preserves its edge-state conductance even at elevated temperatures.^22^ It can be epitaxially grown with atomic precision on H-passivated SiC(0001) substrates (H-SiC)^23,24^ offering a scalable and CMOS-compatible route toward device integration. Moreover, ambient protection of bismuthene has been achieved when it forms via Bi intercalation at the zero-layer graphene (ZLG)/SiC interface.^25,26^ However, the structural properties of such a material system have not yet been fully investigated at the atomic scale.

The defining hallmark of bismuthene as a QSH insulator is the presence of one-dimensional (1D) metallic edge states within the bulk band gap. Although these edge states have been extensively investigated in pristine bismuthene/H-SiC,^23,24^ it remains an open question whether their electronic properties are preserved in bismuthene intercalated beneath ZLG. The graphene overlayer may alter the dielectric environment, electronic screening, and interface coupling, thereby influencing the edge-state properties. Addressing this question is therefore crucial for assessing the robustness of the topological edge states in graphene-capped bismuthene.

In this work, we investigated bismuthene intercalated beneath zero-layer graphene on SiC(0001) using low-temperature scanning tunneling microscopy/spectroscopy (LT-STM/STS), spot-profile analysis low-energy electron diffraction (SPA-LEED), angle-resolved photoemission spectroscopy (ARPES), and density functional theory (DFT) calculations. STM imaging reveals that Bi intercalation leads to the formation of nanoscale bismuthene islands with well-defined armchair edges. Spatially resolved STS measurements show a bulk band gap and metallic edge state. Remarkably, tunneling spectra recorded along the island boundaries reveal a pronounced suppression of the local density of states (LDOS) near the Fermi level, consistent with Tomonaga–Luttinger liquid (TLL) behavior in the one-dimensional edge channels of the intercalated bismuthene layer.

## Results and Discussion

### Structure and Morphology of 2D-Bi Islands

The high-resolution diffraction pattern shown in Figure 1a reveals the characteristic diffraction spots of SiC(0001), graphene, and bismuthene, as well as the $\left(6\sqrt{3}\times 6\sqrt{3}\right)$ Moiré spots of graphene relative to the SiC substrate. The line scans in panel b show not only the first- and higher-order spots of the bismuthene $\left(\sqrt{3}\times \sqrt{3}\right)R30^{{}^{\circ}}$ reconstruction, reminiscent of the formation of well-ordered bismuthene phase, but also the bell-shaped background indicative of a fully delaminated ZLG and its transformation into a quasi-free-standing monolayer graphene.^27–29^

**Figure 1:**
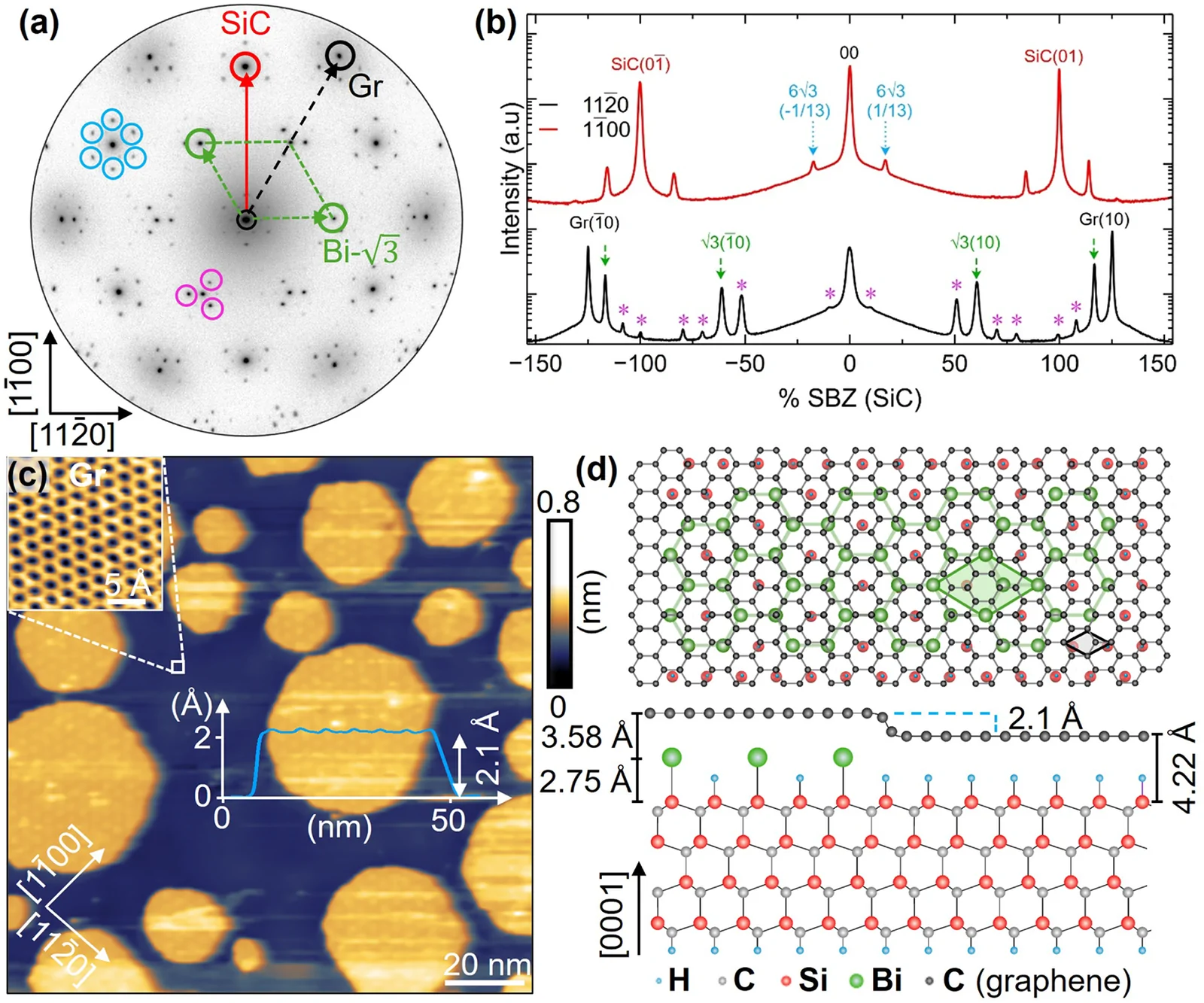
Intercalation and hydrogenation of Bi below ZLG. (a) SPA-LEED pattern taken at E = 167 eV of Bi-intercalated ZLG/SiC showing the graphene (black circle), Bi- $\left(\sqrt{3}\times \sqrt{3}\right)R30^{{}^{\circ}}$ (green circle), SiC (red circle), $\left(6\sqrt{3}\times 6\sqrt{3}\right)R30^{{}^{\circ}}$ (purple circle) and (6 × 6) (blue circle) periodicity. (b) Line profiles along two crystallographic directions. All diffraction experiments were performed at T = 300 K. (c) Large-scale STM topographic image (+1 V, 450 pA, 4.5 K) showing intercalated bismuthene islands (EG/Bi/SiC) surrounded by H-intercalated ZLG area (HQFMLG). Inset: STM image showing atomically resolved HQFMLG recorded at +1.8 V, and 300 pA, T = 4.5 K. The line scan across the bismuthene islands reveals an apparent height of 2.1Å. (d) Ball and stick model of bismuthene- and H-intercalated ZLG on SiC: top view (top) and side view (bottom).

The large-scale STM image (Figure 1c) fully supports these findings and additionally reveals the formation of numerous islands with an average lateral size of around 24 × 22 nm^2^ (SI, Figure S1g). The islands reveal a Moiré structure, arising from the lattice mismatch between the SiC (*a*_SiC_ = 3.08 Å), bismuthene (*a*_Bi_ = 5.32 Å) and graphene (*a*_Gr_ = 2.46 Å). Prior to the formation of the bismuthene phase, a *β*-Bi phase with $-(\sqrt{3}\times \sqrt{3})R30^{{}^{\circ}}$ symmetry is formed. Thereby, Bi atoms occupy *T*_4_ hollow sites of a SiC surface and form three Bi–Si bonds, saturating all Si dangling bonds and preventing planar Bi–Bi hybridization.^26,30,31^ To form finally bismuthene, the *β*-Bi phase was hydrogenated. Hydrogen atoms preferentially passivate Si dangling bonds; thus, the Bi atoms are shifted to the *T*_1_ positions above the Si atoms, leading finally to the formation of bismuthene.^26^ The graphene atop is a quasi-free MLG (BiQFMLG in the following), separated at a distance of 3.58 Å from the intercalated bismuthene layer. In the bismuthene phase, each Bi atom is covalently bonded only to one Si atom of the substrate, thus enabling planar Bi–Bi hybridization and stabilizing the bismuthene honeycomb lattice, as depicted in Figure 1d. Moreover, the hydrogenation process leads to the formation of hydrogen-intercalated areas, revealing a second type of quasi-free-standing monolayer graphene (HQFMLG),^32^ thus stabilizing certain edges of bismuthene islands and ensuring a full carpeting with a graphene layer. This is obvious from the line scan shown in Figure 1c). The apparent height difference of ∼2.1 Å fits nicely to the heights reported for bismuthene and H- intercalated ZLG of ∼6.33 Å and ∼4.22 Å, respectively (SI, Figure S1h).^33,34^

The observation that predominantly islands are formed may provide insights into the coverage of the individual phases. Starting from the *α*-Bi phase, which corresponds to a monolayer coverage, desorption leads to the formation of a *β*-Bi precursor phase. Upon hydrogenation, this phase transforms into bismuthene. The coverage of the bismuthene honeycomb phase corresponds to two-thirds of a monolayer. Assuming that the *β*-Bi phase indeed exhibits a coverage of one-third of a monolayer, as also suggested in the literature,^30^ island formation is indeed to be expected. Compared to previous studies, in which the edges of the 2D QSH system were often located close to SiC substrate steps^23,35,36^ we realized encapsulated bismuthene islands on flat SiC terraces, which facilitates the analysis of the edges and their associated edge states as we will show in the following.

To further corroborate these findings and resolve the sublattices of both 2D heterosystems, the sample was examined by bias-dependent STM measurements. Bismuthene is a topological insulator with a finite bulk band gap, in contrast to the semimetallic character of the graphene overlayer. This distinction allows the lattice of each material layer to be probed selectively by adjusting the bias voltage in STM measurements. Figure 2a–b show constant-current STM images of the same region, where each of the two lattices can be probed by selecting bias voltages. Indeed, at *V*_*b*_ = +0.4 V, the STM image shows only the graphene honeycomb lattice. The Bi honeycomb lattice (bismuthene) appears at a bias voltage of +2 V, mostly probing the states of the bismuthene bulk bands, as also confirmed by STS (see below). The corresponding FFT images are presented in Figure 2a–b as well, revealing the Fourier coefficients of the graphene, bismuthene, and SiC (1 × 1) lattices marked by the black, green, and red circles, respectively. The FFT image in Figure 2a (*V*_*b*_ = +0.4 V) exhibits the intense spots of graphene in addition to the SiC (1 × 1) and Moiré pattern, indicating the formation of QFMLG upon intercalation. Meanwhile, the bismuthene layer is directly probed at *V*_*b*_ = +2 V, giving rise to intense spots of the $\left(\sqrt{3}\times \sqrt{3}\right)R30^{{}^{\circ}}$ lattice in Figure 2b, in good agreement with the SPA-LEED pattern (SI, Figure S1b–d).

**Figure 2:**
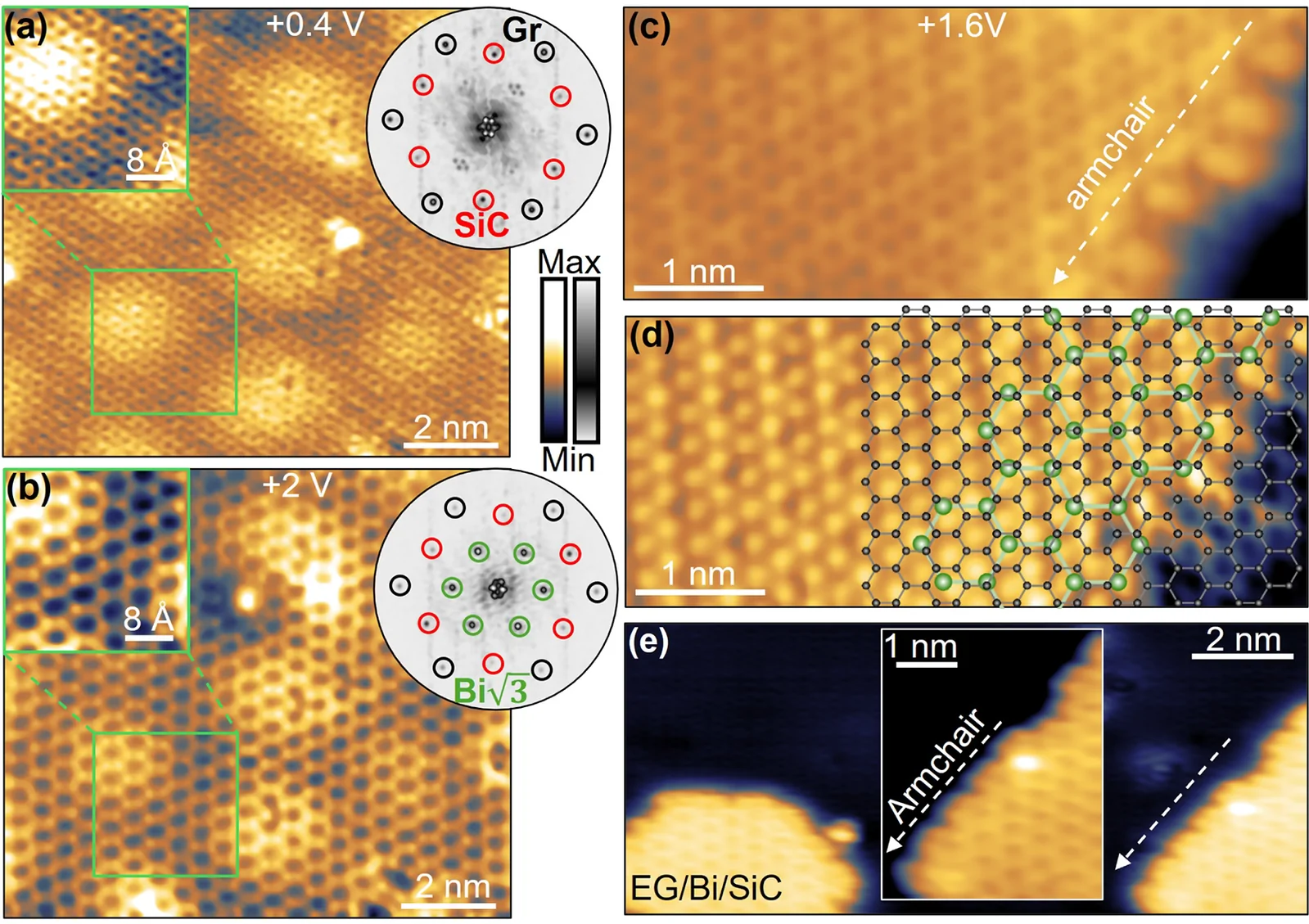
Bismuthene structure and edges. (a–b) STM topographic and FFT images of a bismuthene-intercalated ZLG island measured at two different bias voltages: *V_b_* = +0.4 V (a), and *V_b_* = +2 V (b). Inset: Zoomed-in STM images (left) of the regions outlined by the green rectangles, showing the honeycomb lattice of graphene and bismuthene. The corresponding FFT images are presented in Figure 2a–b (right). (c–d) STM topographic (c) image and current map (d) of an intercalated bismuthene island exhibiting the armchair termination of bismuthene (+1.6 V, 450 pA). Schematic illustration of the graphene (black) and Bi-honeycomb lattice (green) is superimposed on the current mapping image. (e) STM topography of a small region exhibiting two intercalated bismuthene islands (EG/Bi/SiC) (+3 V, 250 pA). Inset: Zoomed-in STM image of the armchair edge. T = 4.5 K.

In the following, we focus on elucidating the edge structure of the intercalated bismuthene islands. This interpretation is supported by the atomic-resolution STM image (Figure 2c–e), acquired by scanning over a small region. The STM image in Figure 2c reveals the interface structure of the island, where both lattices are resolved at the atomic scale. Remarkably, the island boundary corresponds to an armchair termination of the intercalated bismuthene layer.^37^ The graphene overlayer remains continuous across the armchair-terminated boundary (SI, Figure S2a), exposing alternating Bi dimers at the edge, as evidenced by the current mapping image in Figure 2d). This observation agrees well with the STM topography in Figure 2e, where the alternating Bi dimers are clearly observed at the armchair edge.

For bismuthene islands grown on SiC(0001), predominantly armchair-type edges are observed, even in the intercalated case.^23,35^ This preference has been attributed to step-induced edge formation on the SiC substrate.^24^ In our case, the intercalated bismuthene islands exhibit armchair (≈73%) and zigzag (≈27%) edges, regardless of the presence of SiC step edges (SI, Figure S2e), suggesting an intrinsic energetic preference. The measured periodicity along the armchair edges, $\sqrt{3}a_{Bi}=3a_{SiC}=9.24\;Å$ , agrees with the expected lattice match, excluding relaxation effects. The armchair edges remain well-defined after hydrogen post-treatment, as observed in our STM images (SI, Figure S2b–c). Notably, such edge terminations have been shown to influence the electronic structure of the associated topological edge states.^38^

### Electronic Bulk Properties of Bismuthene under Cover

Recent ARPES results showed that the graphene overlayer interacts only weakly with the underlying bismuthene layer, acting primarily as a chemically inert and protective cap.^25,26^ This weak interaction suggests that the graphene layer does not significantly modify the intrinsic electronic structure of the intercalated bismuthene layer. Motivated by this picture, we first examine the bulk electronic structure of bismuthene on an H-passivated SiC substrate using DFT calculations (SI, Figure S6a). As shown in Figure 3a, the valence band (VB) near the Fermi level, primarily composed of Bi *p*_*x*_/*p*_*y*_ orbitals, splits into two bands (*S*_1_ and *S*_2_) with an energy separation of 0.37 eV at the *K* point induced by the strong spin–orbit coupling of Bi and the interface inversion symmetry breaking (Rashba effect) induced by the substrate.^23^ In accordance with the van-der-Waals type interaction between graphene and bismuthene, the gap is slightly lower than the value of 0.485 eV calculated for both one-sided inversion-symmetry broken bismuthene and for bismuthane, where both sides are covalently functionalized.^38^ Notably, the *S*_1_ band lies slightly above the Fermi level as a result of substrate-induced p-doping, consistent with ARPES measurements (SI, Figure S8).^26^ Furthermore, the conduction-band minimum at the Γ point gives rise to an indirect band gap of 0.54 eV. DFT calculations for the heterostructure including the graphene layer were also performed using an approximate (2 × 2) graphene supercell on bismuthene/ $\left(\sqrt{3}\times \sqrt{3}\right)$ -SiC (SI, Figure S6b–e). The results reveal n-type doping of graphene, which could be related to charge transfer from the intercalated bismuthene layer and possibly the SiC substrate (SI, Figure S8),^25^ indicating weak van der Waals interactions between the layers.

**Figure 3:**
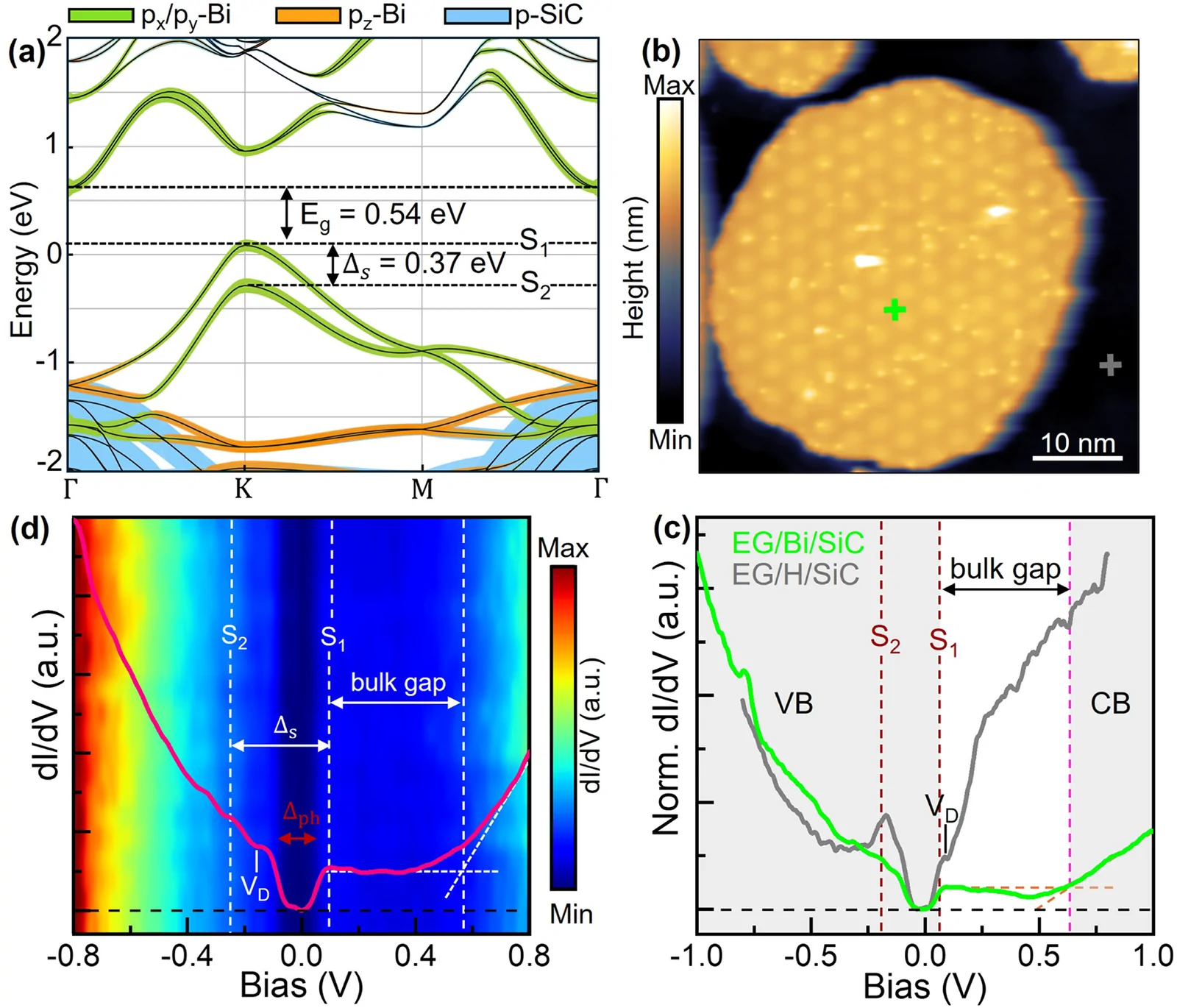
Bulk electronic structure of bismuthene. (a) DFT calculations for the band structure of bismuthene/H-passivated SiC. The indirect band gap of bismuthene is denoted as *E_g_*, while Δ*_s_* represents the energy spacing between the *S*_1_ and *S*_2_ bands of the valence band (VB). (b) STM topography of intercalated bismuthene islands (EG/Bi/SiC) surrounded by an H-intercalated ZLG region (EG/H/SiC) (+0.8 V, 450 pA). (c) Tunneling *dI*/*dV* spectra (STS) recorded at two different positions marked by the green and gray crosses as indicated in Figure 3b. Tunneling conditions are *V_b_* = +1 V (green), *V_b_* = +0.8 V (grey), *I_t_* = 450 pA. Two tangential lines (orange) were drawn to the green STS curve to determine the conduction band (CB) edge. The Dirac point is denoted as *V_D_*. (d) Average *dI*/*dV* spectrum measured at the island interior (green cross) on the top of 20 consecutive *dI*/*dV* curves concatenated into a color map (*V_b_* = +0.8 V, *I_t_* = 450 pA). In the STS map, *S*_1_ and *S*_2_ represent the top and next energy levels of the VB, respectively. The black dashed lines represent the zero dI/dV intensity. T = 4.5 K.

To further elucidate the electronic structure, scanning tunneling spectroscopy (STS) was employed to probe the local density of states (LDOS) of the intercalated bismuthene islands. Figure 3c shows representative *dI*/*dV* spectra acquired at the island interior (Figure 3b, green cross) and on the H-intercalated ZLG region (Figure 3b, gray cross). For EG/Bi/SiC, the spectrum measured on the island (green) displays distinct features, including the upper valence band state (*S*_1_) at +0.1 V and a nearly constant DOS region above *S*_1_. The onset near +0.5 eV corresponds to the bulk conduction band of bismuthene and agrees well with DFT calculations (SI, Figure S7). The DOS within this gap, however, is not completely suppressed, reflecting the contribution of the overlying graphene layer. The STS curve reveals a suppression of the DOS at *E*_*F*_, accompanied by an apparent gap. This characteristic feature arises from inelastic tunneling processes mediated by a phonon that supplies the necessary momentum for transitions into the graphene *π*-states at the K-point.^39^ The observation of this gap is a well-established hallmark of freestanding, structurally intact graphene. The slight asymmetry of the graphene-related phonon gap in the bulk spectra originates from the contribution of the bismuthene valence band states in this energy range, in agreement with observations reported for other intercalated systems.^40^ We want to point out that a quantitative calculation of the density of states and the corresponding *dI*/*dV* spectra is unfortunately not available at this stage. Consequently, our interpretation is based on a qualitative assignment guided by previous results. For this specific material system, we first matched the spectral features observed at the bulk position with the electronic structure obtained from ARPES measurements and DFT calculations. Using this assignment as a reference, we subsequently analyzed the spectral modifications occurring toward the edges.

The results presented in Figure 3d show a total of 20 spectra taken at an interior position of the intercalated bismuthene island, demonstrating the reproducibility and robustness of the above-discussed spectral fingerprints. The pink curve represents the averaged spectrum, enabling a clearer identification of the *S*_1_ and *S*_2_ states, as well as the splitting energy (Δ_*s*_ = 0.34 eV), in good agreement with the DFT calculations. The dip at −0.16 V is attributed to the Dirac point (*V*_*D*_) for the n-doped graphene on top of the bismuthene layer (SI, Figure S8).^26^

We also measured the *dI*/*dV* curve obtained on HQFMLG (Figure 3c, gray curve). Apart from the inelastic tunneling gap, which is also expected for HQFMLG, the spectrum exhibits distinct features. In particular, the small dip around +95 mV can be associated with the Dirac point *V*_*D*_.^41^ The observed *p*-type doping for the only H-intercalated ZLG areas is consistent with the ARPES results (SI, Figure S8).^26,32^

### Island Size and Edge Effects of Bismuthene Islands

We have shown above a weak electronic coupling between graphene and bismuthene, allowing us to study the intrinsic electronic structure of the intercalated bismuthene layer. The formation of islands with different sizes naturally raises the question of how this affects the bulk electronic bands of the intercalated bismuthene. The *dI*/*dV* spectra (STS) shown in Figure 4a) for three different islands display very similar spectral features. Although the local DOS varies slightly, no significant modifications are observed in the band structure measured at the interior of each island. This indicates that the bulk band gap of bismuthene remains essentially insensitive to the island’s lateral dimensions, as expected for 2D topological insulators.^42^

**Figure 4:**
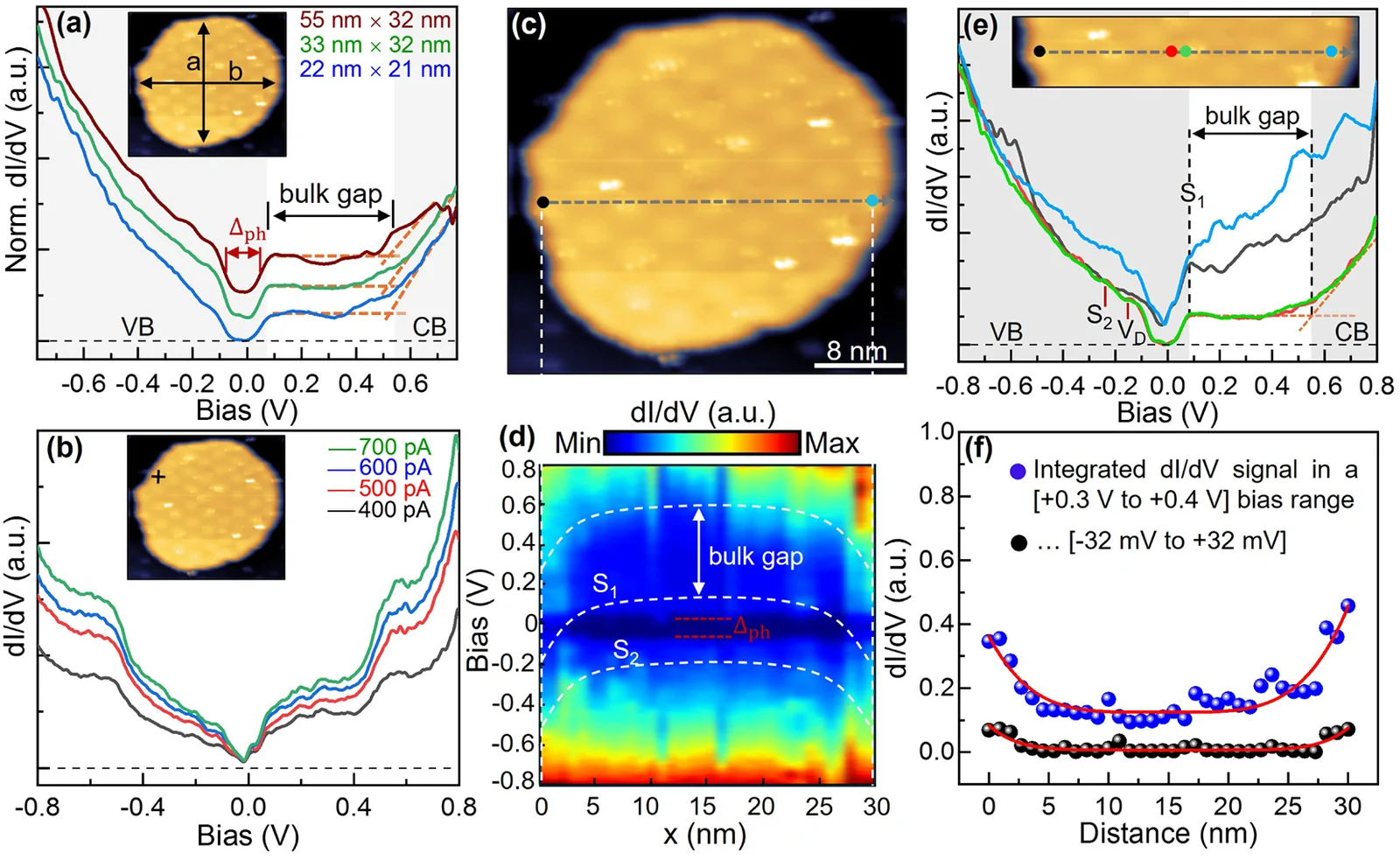
Size- and side-effects of bismuthene islands. (a) Normalized *dI*/*dV* spectra of three intercalated bismuthene islands with different lateral sizes (a, b). The spectra were acquired at an interior position of each island. Island 1 (22 × 21 nm^2^): *V_b_* = 0.8 V, *I_t_* = 500 pA. Island 2 (33 × 32 nm^2^): *V_b_* = 0.8 V, *I_t_* = 450 pA. Island 3 (55 × 32 nm^2^): *V_b_* = 0.8 V, *I_t_* = 370 pA. The spectra are shifted for better visibility. (b) Set-point current-dependent STS recorded at the position marked by the black cross (inset image) (*V_b_* = +0.8 V). (c) STM topography of an intercalated bismuthene island measured at +1.5 V and 400 pA. (d) *dI*/*dV* map of spectra (*V_b_* = +0.8 V, *I_t_* = 450 pA) measured along the grey dashed arrow shown in Figure 4c. The phonon-induced gap is denoted by Δ_ph_. The dashed (white) lines are a guide to the eyes to illustrate the band bending (see also SI). (e) Representative *dI*/*dV* spectra were extracted from the positions marked by the colored circles, shown in the inset image (cut off from Figure 4c). Two tangential lines (orange) were drawn to the green and red STS curves to determine the CB edge. (f) Integrated *dI*/*dV* signal in a [+0.3 V to +0.4 V] bias range (within the bulk gap, blue) and a [−32 mV to +32 mV] bias range (within the phonon-induced gap, black). The data points were multiplied by 10 for better visualization. The fitted curves (red) were obtained using a power-law function. The black dashed lines represent the zero dI/dV intensity. T = 4.5 K.

While the spectra acquired at the island interior exhibit a constant-like DOS region indicative of the bismuthene-derived bulk gap, the STS measured at the island boundary displays a V-shaped DOS with enhanced spectral weight on both applied bias sides (Figure 4b). Moreover, the spectrum also shows a finite DOS at zero bias, reflecting the metallic nature of the bismuthene’s edge states. Figure 4e shows representative spectra in direct comparison taken at the bulk and edge positions. These *dI*/*dV* results unambiguously demonstrate the presence of metallic edge channels in the intercalated bismuthene islands, indicating that the local edge-state electronic structure is largely preserved, despite encapsulation by the graphene overlayer.^23,35^

To better understand the edge behavior relative to the bulk, STS spectra were acquired at 1 nm intervals along the diameter of the bismuthene islands and displayed as a DOS intensity map shown in Figure 4d. In the center region, we mainly find the valence band maximum and conduction band minimum as discussed in the context of Figure 3d. Remarkably, the bismuthene band edge energies gradually decrease toward the edges of the bismuthene island, pointing toward a band bending effect indicated by the dashed lines. The phonon-induced gap of graphene appears to persist but is superimposed by a finite intensity within the former gap region (Figure 4d). A similar result was obtained at 77 K (see SI, Figure S4).

To better illustrate the spatial evolution of the local density of states, we compared the position-dependent intensity of two characteristic energy ranges. Specifically, we focused on the energy region around the Fermi level and the conduction band edge of bismuthene, representing the edge channel and the bulk band, respectively. As clearly shown in Figure 4f, the integrated intensity of both signals increases toward the edge. This increase can be attributed to the presence of the metallic edge channel as well as to the band bending of the bulk bands.

### Edge States along Bismuthene Islands

We next examine *dI*/*dV* conductance maps of a distinct armchair edge, whose morphology and site-resolved electronic structure are shown in Figure 5a and b. Figure 5c–d present the constant-current STM image alongside the corresponding *dI*/*dV* map. The STM image reveals an enhanced tunneling signal with a characteristic periodicity of $\sqrt{3}a_{\mathrm{Bi}}=0.92\;\mathrm{nm}$ , consistent with armchair edges in pristine bismuthene.^35^ In the *dI*/*dV* map, a pronounced and spatially localized enhancement of tunneling conductance is observed along the edge, in stark contrast to the suppressed conductance within the island interior (white arrow) and the HQFMLG region (blue arrow). The conductance map was acquired at +0.5 V, such that the intensity distribution contains contributions from both the edge states and the bulk bands, as already discussed in the context of Figure 4f. Conductance maps recorded close to the Fermi level did not provide reliable information, most likely due to the continuous overgrowth of the island edges, which prevents a stable spectroscopic characterization of the edge-state contribution for such small tip-sample distances.

**Figure 5:**
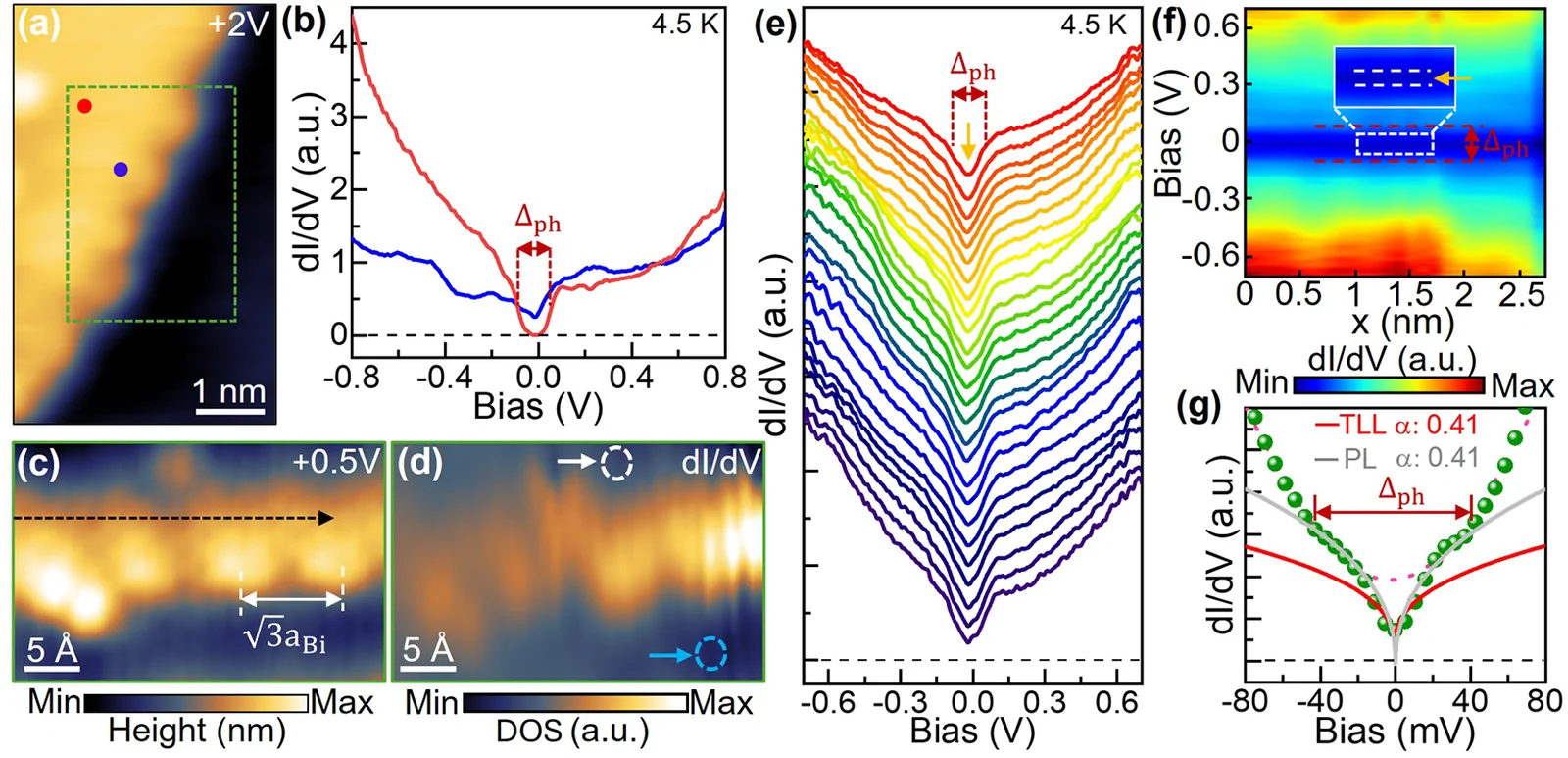
Spectroscopy of the edge states. (a) STM topography of an intercalated bismuthene island boundary (+2 V, 450 pA, 4.5 K). (b) Tunneling *dI*/*dV* spectra (STS) recorded at two different positions marked by the red and blue circles in Figure 5a. (c) STM topography and conductance map (d) of a small region outlined by the green dashed rectangle in Figure 5a measured at +0.5 V and 420 pA. The white and blue arrows indicate the island interior and EG/H/SiC, respectively. (e) Stacked *dI*/*dV* spectra taken along the direction marked in panel (c). (f) Corresponding STS map. Inset: Zoomed-in image showing the small LDOS dip (orange arrow) at *E_F_*. (g) High-resolution edge spectrum (green) showing both the phonon-induced gap *Δ_ph_* in graphene (pink dashed curve) in addition to signatures of the bismuthene edge state. The latter can be rationalized using the TLL model (red) and the power-law (PL) function (∼|*V*_b_|*^α^*, gray) with *α* = 0.41.^35^ The black dashed lines represent the zero dI/dV intensity. All dI/dV spectra measured at *V_b_* = +0.8 V, *I_t_* = 450 pA, T = 4.5 K.

To further characterize the edge states, tunneling spectra were measured at different positions along the investigated armchair edge (black dashed arrow in Figure 5c. As is obvious from Figure 5f, the STS map reveals a nearly uniform spectral feature with a pronounced LDOS enhancement, indicating that the metallic edge state is continuously distributed along the armchair edge. The corresponding spectra (Figure 5e) exhibit the characteristic V-shaped line shape with a finite offset at *E*_*F*_ and the phonon-induced band gap feature in graphene *Δ*_*ph*_, in agreement with data shown before. These findings are consistent with earlier DFT calculations on SiC-adsorbed bismuthene (and bismuthane) nanoribbons, which report a topologically protected Dirac-cone edge channel inside the planar bismuthene band gap and already negligible finite size effects in a 4 a_0_ wide nanoribbon.^38^

The high reproducibility of the spectra is further demonstrated by the STS map shown in panel (f). Most notably, a subtle substructure at the Fermi level within the phonon-induced gap is consistently observed across the entire map. The zoomed-in view further highlights this feature (the orange arrow). Panel (g) presents a representative low-energy spectrum, where the phonon-induced gap, marked by the pink dashed curve, is clearly resolved. In addition, a distinct dip at the Fermi level, commonly referred to as a zero-bias anomaly (ZBA), is observed within a narrow bias range.

Of course, such ZBA fingerprints can arise from a variety of underlying mechanisms, including Efros–Shklovskii (ES) pseudogap,^43^ Coulomb blockade^44,45^ or Tomonaga-Luttinger liquid (TLL) formation.^35^ In our case, however, the observed STS data are not consistent with the Coulomb blockade scenario. Specifically, the low-energy spectral feature remains essentially unchanged over a range of tunneling currents, indicating that it is not dominated by tip-induced charging effects (see SI, Figure S5e).^35^ Furthermore, the spectra do not exhibit the discrete charging features typically associated with Coulomb blockade under the investigated range of tunneling conditions and temperatures (SI, Figure S5a–e).^46^ Likewise, the edge spectra in Figure 5g exhibit a power-law suppression of the LDOS within a narrow bias range, inconsistent with the exponential suppression expected for the ES pseudogap.^43^

Because of the overlap of the two spectral features at the Fermi level, a reliable analysis of the microscopic origin of the zero-bias anomaly (ZBA) is challenging at this point. We therefore refer to previous analysis of uncapped bismuthene islands, where the larger accessible energy window and the availability of temperature-dependent measurements provided a substantially more robust basis for the analysis.^23,35,47^ Applying the same approach to the graphene-capped bismuthene yields satisfactory agreement. In particular, fitting the spectral suppression with a power-law dependence ∝ |*E* – *E*_F_|^*α*^ using *α* = 0.41, provides a reasonable description of the finite-bias conductance. This behavior is consistent with a deviation from conventional metallic Fermi-liquid behavior. We further applied the standard theoretical framework for correlated one-dimensional metallic systems, namely the Tomonaga–Luttinger liquid (TLL) model, which explicitly accounts for the temperature dependence of the tunneling spectra.^35,48^ The corresponding curve with the same *α* value, shown in Figure 5g, is likewise consistent with our experimental data and supports the interpretation that electronic correlation effects persist in the one-dimensional edge channels of bismuthene. These results further suggest that the correlated edge-state behavior remains preserved despite the graphene capping layer.

## Conclusions

In summary, we have investigated the structural and electronic properties of bismuthene intercalated beneath zerolayer graphene (ZLG) by combining low-temperature STM/STS, SPA-LEED, and density functional theory (DFT) calculations. The intercalation gives rise to a well-ordered bismuthene lattice beneath epitaxial graphene with well-defined edge terminations, particularly armchair-oriented edges. Spatially resolved STS measurements demonstrate that the island interior exhibits a large bulk band gap of approximately 0.5 eV, in excellent agreement with the DFT-predicted electronic structure. Toward the edges, we observe band bending of the bulk bands and the emergence of in-gap edge states, evidenced by a finite zero-bias conductance. A detailed analysis of the one-dimensional armchair edges of the bismuthene islands revealed that, in addition to the characteristic phonon gap originating from graphene, a second dip of the LDOS around *E*_*F*_ is present. A similar spectral feature has been observed for bismuthene on H-passivated SiC, where it was interpreted as the signature of a Tomonaga–Luttinger liquid (TLL).^23,35^ Our data are consistent with this interpretation, indicating that the characteristic TLL-like spectral feature is preserved despite the graphene overlayer. Although the enhanced LDOS observed at the edges does not by itself constitute direct proof of topological protection, the combined structural and spectroscopic evidence—including the well-defined armchair termination, the bulk gap consistent with DFT calculations and the characteristic metallic edge states—is consistent with topological edge states of bismuthene.^23,35^ In this context, graphene not only protects the bismuthene layer from environmental influences but also preserves its intrinsic topological edge states,^25^ providing a versatile platform for studying quantum transport and many-body physics in one-dimensional topological edge channels.

## Materials and Methods

### Sample Preparation

ZLG was epitaxially grown on a SiC(0001) substrate using the polymer-assisted sublimation growth, as described in detail elsewhere.^49,50^ Following initial degassing and characterization, Bi deposition was performed in a dedicated UHV chamber with a base pressure of less than 1 × 10^–6^ Pa by evaporation from a Knudsen cell kept at 550 °C for 120 min. After transferring the sample to the analysis chamber (base pressure <2 × 10^–8^ Pa), Bi intercalation was achieved by annealing at 450 °C for 30 min. The sample temperature was monitored with an infrared pyrometer (emissivity 0.9). Transformation to the *β*-phase was achieved by Bi dilution at elevated temperatures (950 °C for 10 min), as described in previous reports.^30,31^ Bismuthene formation was accomplished by annealing the sample at 550 °C for 90 min in a dedicated contactless infrared heating system under 850 mbar of H_2_ at a flow rate of 0.9 slm. Prior to STM measurements, the sample quality was verified through photoelectron spectroscopy, in comparison with previous studies.^25,26^

### LT-STM and SPA-LEED Measurements

After intercalation, the samples were immediately transferred via a UHV suitcase to the SPA-LEED system to examine the overall sample quality (*p* = 10^–9^ Pa). The high-resolution diffraction profiles allow for the determination of surface roughness and the presence of graphene. Subsequently, the samples were transferred in situ into the STM chamber, where their structural and electronic properties were investigated at low temperatures (4.5 and 77 K) under UHV conditions. PtIr tips were calibrated on Au(111) substrates prior to STM/STS measurements. STM measurements were carried out in constant-current mode. The feedback loop was turned off while recording the *dI*/*dV* maps. Tunneling *dI*/*dV* spectroscopy (STS) was performed in constant-height mode using a lock-in amplifier with a modulation voltage of *V*_rms_ = 10 mV and 12 mV and modulation frequencies of *f*_rms_ = 860 and 750 Hz.

### Angle-Resolved Photoemission Spectroscopy (ARPES)

The samples were transferred into a UHV chamber dedicated to ARPES measurements (*p* = 3 × 10^–8^ Pa). The chamber was equipped with a monochromatized SPECS Focus 500 Al–K*α* X-ray source, a monochromatized SPECS UVS 300 ultraviolet light source providing linear polarized He–I and He–II radiation, and a SPECS Phoibos 150 hemispherical electron analyzer with the 2D-CCD detector.

### Computational Details

The structural relaxations of the initial configurations and the calculation of the electronic properties, including the band structure and the density of states (DOS), were performed within density functional theory (DFT) as implemented in the ABINIT package.^51,52^ The exchange–correlation potential was treated within the generalized gradient approximation using the Perdew–Burke–Ernzerhof (PBE) functional.^53^ Fully relativistic norm-conserving pseudopotentials were employed throughout. Structural relaxations were carried out using an 8 × 8 × 1 Monkhorst–Pack *k*-point mesh until all atomic forces were below 0.0025 eV/Å. Dispersion interactions between layers were taken into account using the D3 van der Waals correction scheme.^54^ For the self-consistent calculations, a total energy convergence threshold of 2.7 × 10^–8^ eV and a plane-wave kinetic energy cutoff of 1088 eV were used.

The intercalated graphene structure was modeled using a commensurate supercell approach, in which a (2 × 2) graphene supercell corresponds to a $\left(\sqrt{3}\times \sqrt{3}\right)$ SiC supercell. This commensurability was achieved by applying an in-plane strain of approximately 8% to the graphene layer.^55,56^ At the interface between graphene and the SiC substrate, a bismuthene monolayer with $\left(\sqrt{3}\times \sqrt{3}\right)$ -SiC periodicity was introduced.^23,29^ In this configuration, two of the three Si atoms in the topmost SiC surface layer were saturated with Bi atoms, while the remaining Si atom was passivated with hydrogen. The dangling bonds at the bottom surface of the SiC substrate were also passivated with hydrogen atoms. A vacuum spacing of 15 Å was included along the out-of-plane direction to avoid spurious interactions between periodic images.

## Supplementary Material



## Data Availability

The data that support the findings of this study are available from the corresponding author upon reasonable request.

## Notes

Details of the STM and STS of graphene with intercalated bismuthene; STM analysis of the edges; details of the DFT calculations; additional ARPES measurements (PDF)
