# Supplementary material for: Protected Metallic Edge States in a Graphene-Capped Quantum Spin Hall System
Source: ACS Nano. 2026 Sep 30;20(40):27472–81. doi: 10.1021/acsnano.6c14197 (PMC13671255; doi:10.1021/acsnano.6c14197)
Supplement: Supplementary file 1 [file nn6c14197_si_001.pdf]

# Supporting Information: Protected metallic edge states in a graphene-capped quantum spin Hall system

Huu Thoai Ngo,[†] Zamin Mamiyev,[†] Niclas Tilgner,[†,‡] Andres David Peña Unigarro,[†,‡] Sibylle Gemming,[†,‡] Thomas Seyller,[†,‡] and Christoph Tegenkamp[*,†,¶]

†*Institut für Physik, Technische Universität Chemnitz, Reichenhainer Str. 70, D-09126 Chemnitz, Germany*

‡*Research Center for Materials, Architectures and Integration of Nanomembranes (MAIN), Chemnitz, Germany*

E-mail: christoph.tegenkamp@physik.tu-chemnitz.de

## Supporting information: STM and STS Data

Fig. S1a) shows a topographic STM image of two intercalated bismuthene islands (EG/Bi/SiC) surrounded by a hydrogen-intercalated ZLG on SiC (EG/H/SiC). The coexistence of H- and Bi-intercalated BL/SiC regions leads to a distinct topographic contrast, as illustrated by the height profile in Fig. S1h). The apparent height difference between the EG/Bi/SiC and EG/H/SiC regions is approximately 2.2Å, while the SiC step height is determined to be 2.4Å(Fig. S1h).[S1]

Zoomed-in STM images of the EG/Bi/SiC island (outlined by the green dashed rectangle) and the EG/H/SiC region (outlined by the black dashed rectangle) are shown in Figs. S1b)

and d), respectively. Fig. S1b) reveals the honeycomb lattice of bismuthene with a lattice constant of 5.35 Å, giving rise to the Bi-$(\sqrt{3} \times \sqrt{3})R30^{\circ}$ spots in the corresponding FFT image (Fig. S1c). In contrast, the H-intercalated ZLG region (Fig. S1d) exhibits only the honeycomb lattice of graphene, as confirmed by its FFT image (Fig. S1e). This observation evidences the formation of quasi-free-standing monolayer graphene (HQFMLG).

The close-up STM image of the intercalated bismuthene island (outlined by the red dashed rectangle) clearly displays the armchair termination of the bismuthene layer, with distinct segments marked by white arrows.[S2] Fig. S1g) presents the statistical distribution of the lateral dimensions (a, b) of intercalated bismuthene islands, obtained from measurements of 100 islands.

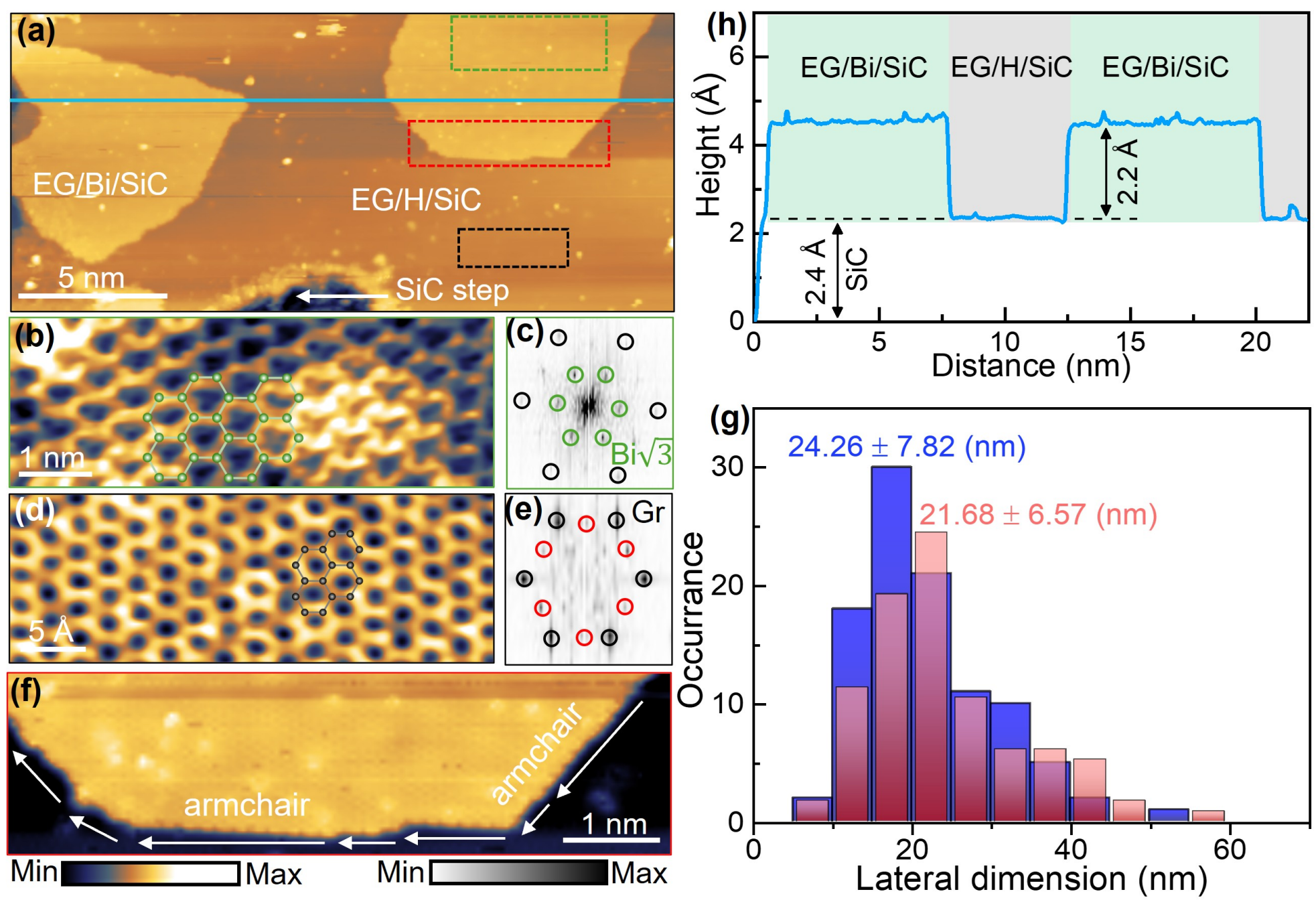


Figure S1: (a) STM topography of a small sample area showing two intercalated bismuthene islands (EG/Bi/SiC) surrounded by H-intercalated ZLG (EG/H/SiC) (+2 V, 300 pA). (b-c) Zoomed-in STM and corresponding FFT images of the region outlined by the green dashed rectangle (+2 V, 250 pA) in Fig. S1a). (d-e) Zoomed-in STM and corresponding FFT images of the region outlined by the black dashed rectangle (+2.5 V, 300 pA) in Fig. S1a). (f) Zoomed-in STM image of the region outlined by the red dashed rectangle in Fig. S1a), displaying the armchair termination of the bismuthene layer with different segments (marked by the white arrows). (h) Height profile extracted from the blue line in Fig. S1a). (g) Statistics of lateral dimension calculated from one hundred intercalated bismuthene islands measured on different samples. The STM images were acquired at 77 K.

Figs. S2a)–b) display STM images of an intercalated bismuthene island, providing insight into the edge structure of the underlying bismuthene layer. As shown in Fig. S2b), the island boundary exhibits both zigzag and armchair terminations of the intercalated layer, while the graphene overlayer grows continuously (Fig. S2a, inset STM image). Similarly, the STM image in Fig. S2c) shows armchair and zigzag edges of the intercalated layer with different lengths. In particular, the armchair edge is observed with an enhanced tunneling current signal and a characteristic $\sqrt{3}a_{\mathrm{Bi}}$ periodicity (9.24Å).[S3] The coexistence of armchair and zigzag terminations is illustrated in the ball model (Fig. S2d). Fig. S2e) presents statistics on the number of zigzag and armchair edges observed from ten intercalated bismuthene islands.

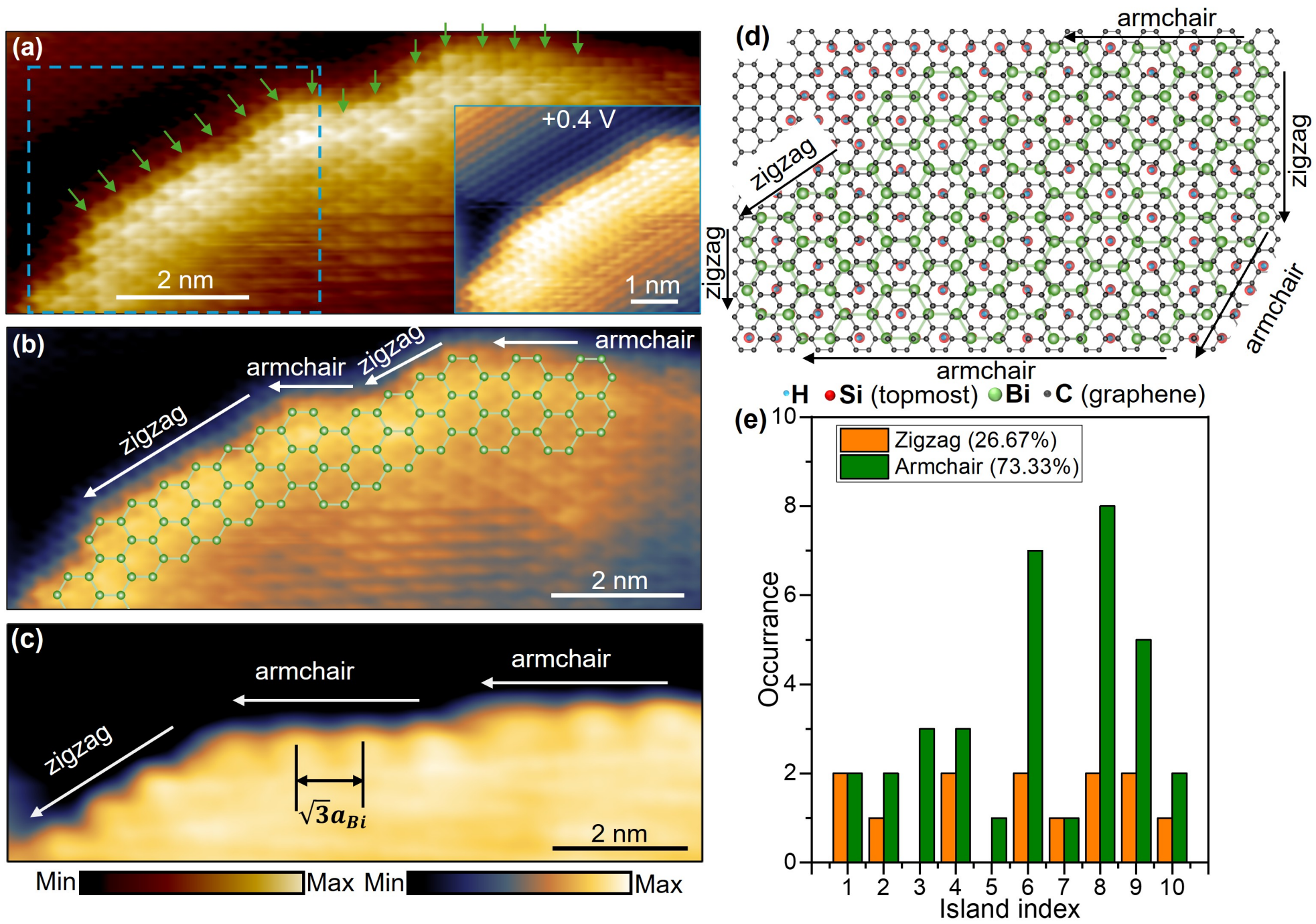


Figure S2: (a-b) STM topography of an intercalated bismuthene island showing the zigzag and armchair termination of the bismuthene film beneath graphene (+2 V, 440 pA): (a) unfiltered and (b) filtered images. Inset: Atomically resolved STM image of a region outlined by a blue dashed rectangle in Fig. S2a), showing the continuous graphene layer across the island boundaries (+0.4 V, 430 pA). (c) STM topography of another intercalated bismuthene island (+1 V, 430 pA), exhibiting mainly armchair edges with the characteristic $\sqrt{3}\,a_{\rm Bi}$ symmetry. (d) Ball-stick model of an intercalated bismuthene island, exhibiting the zigzag and armchair edges. (e) Statistics of the quantity of the observed zigzag and armchair edges from ten of the investigated intercalated bismuthene islands. The STM images were acquired at 4.5 K.

Fig. S3a) shows the STM topography of the intercalated bismuthene island (EG/Bi/SiC), which has been presented in the main text. Figs. S3b)–c) present STM images of an island boundary (the red rectangle in Fig. S3a) acquired at two different bias voltages. In particular, the honeycomb lattice of the graphene overlayer is clearly resolved at $V_b = +0.1$ V (Fig. S3b), whereas the edge structure of the intercalated bismuthene layer becomes visible at +1.5 V (Fig. S3c). The STM image in Fig. S3d) was obtained by scanning a smaller region (the green rectangle) of the island, revealing both graphene and bismuthene lattices probed at $V_b$ = +2 V. Fig. S3e) presents the $dI/dV$ spectra (STS) recorded at different positions marked by colored circles, as indicated in the inset STM image. The spectra measured at bulk positions (black, red, blue) reveal a bulk band gap of the intercalated bismuthene layer, along with a pronounced suppression of the LDOS around the Fermi level—well known as the phonon-induced gap of graphene.[S4] In contrast, the STS recorded at the island boundary (purple) exhibits a V-shaped DOS with a small dip around the Fermi level. Notably, the overall conductance is higher than that of the STS recorded at the bulk positions, indicating the metallic nature of the edge states arising from the underlying bismuthene. These STS results demonstrate that the edge-state electronic structure is largely preserved beneath the graphene overlayer.

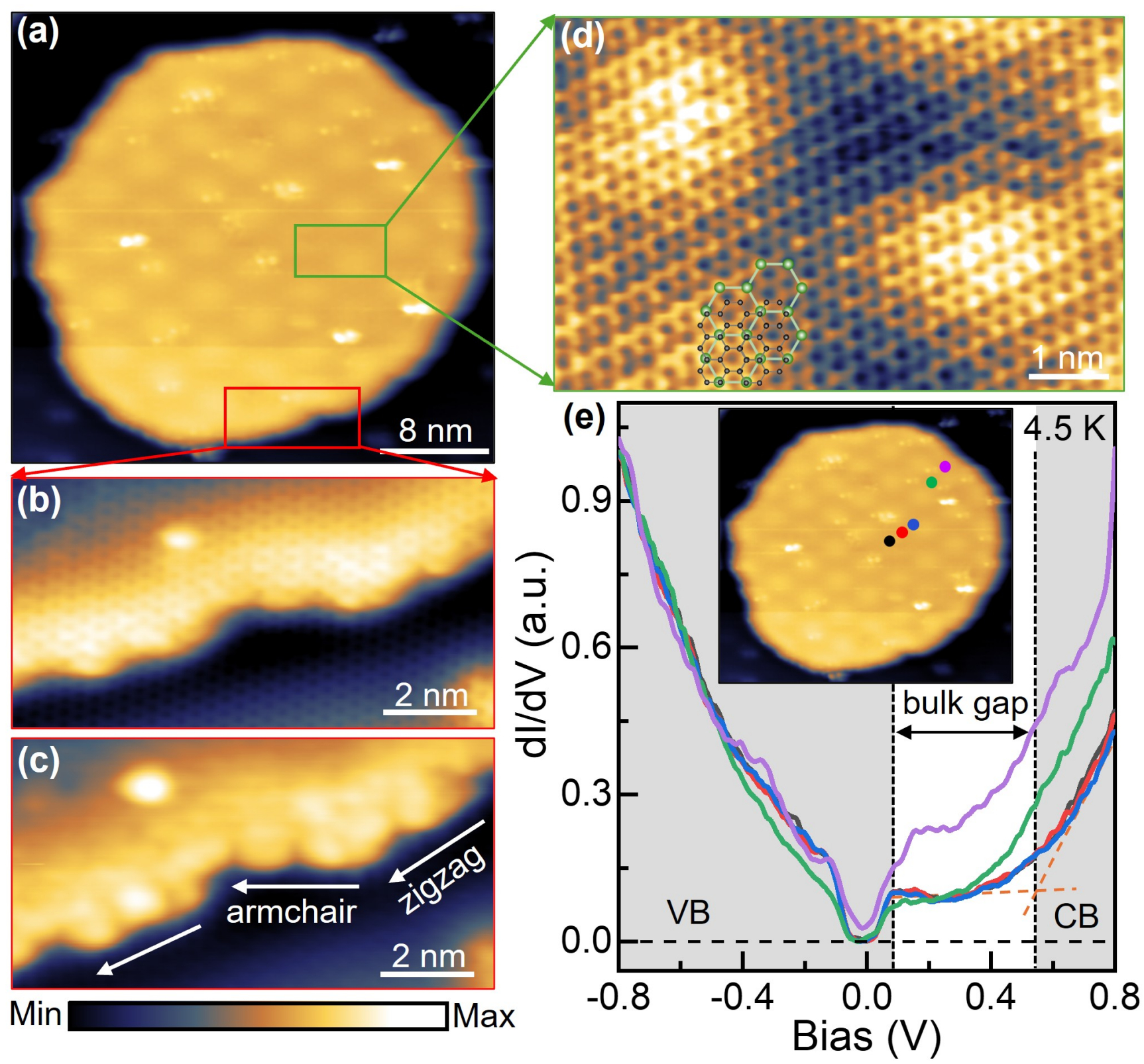


Figure S3: (a) STM topography of an intercalated bismuthene island (+1.5 V, 400 pA). (b-c) STM image of the region outlined by the red rectangle measured at (b) +0.1 V and (c) +1.5 V, showing the zigzag and armchair termination of the intercalated bismuthene layer. (d) STM image of the region outlined by the green rectangle (+2 V, 450 pA). (e) Tunneling spectra recorded at random positions marked by colored circles, as shown in the inserted STM image. The spectra were measured at $V_b = +0.8$ V, $I_t = 450$ pA, T = 4.5 K.

Figs. S4a)–c) show STM and STS results acquired at 77 K. The STM image in Fig. S4a) reveals an intercalated bismuthene island with well-defined boundaries. To investigate the electronic structure of this island, tunneling spectroscopy was performed at different positions along the grey dashed arrow, as indicated in Fig. S4a). The STS map reflects the spatial variation of the LDOS across the island; in particular, a strongly suppressed DOS region is observed above the Fermi level, indicative of the insulating gap of the intercalated bismuthene layer (Fig. S4b). To further interpret the STS map, representative $dI/dV$ spectra from selected positions (colored circles) are shown in Fig. S4c). At the interior positions (red and green circles), the spectra exhibit a pronounced DOS suppression above $E_F$, corresponding to the bulk band gap of the bismuthene layer. This suppressed DOS region vanishes as the tip moves toward the island boundary (blue, purple, and black circles). Remarkably, the $dI/dV$ spectra at the boundary display higher intensity than those recorded in the island interior (red and green circles), providing evidence for the metallic edge state of the intercalated bismuthene layer despite the presence of the graphene overlayer. This interpretation is supported by the data in Fig. S4d), where the integrated $dI/dV$ signal (within the bulk gap) of the island boundaries is higher than that of the island interior. Fig. S4e) displays the set-point current-dependent STS recorded at a position (black cross) near the island boundary. As expected, the overall $dI/dV$ signal increases at higher currents due to the enhanced probability of tunnelled electrons, implying the robust electronic states of the underlying bismuthene film. These findings suggest that bismuthene-intercalated ZLG is a promising material platform for realizing the quantum spin Hall (QSH) effect while simultaneously providing effective protection of the topological layer against oxidation.

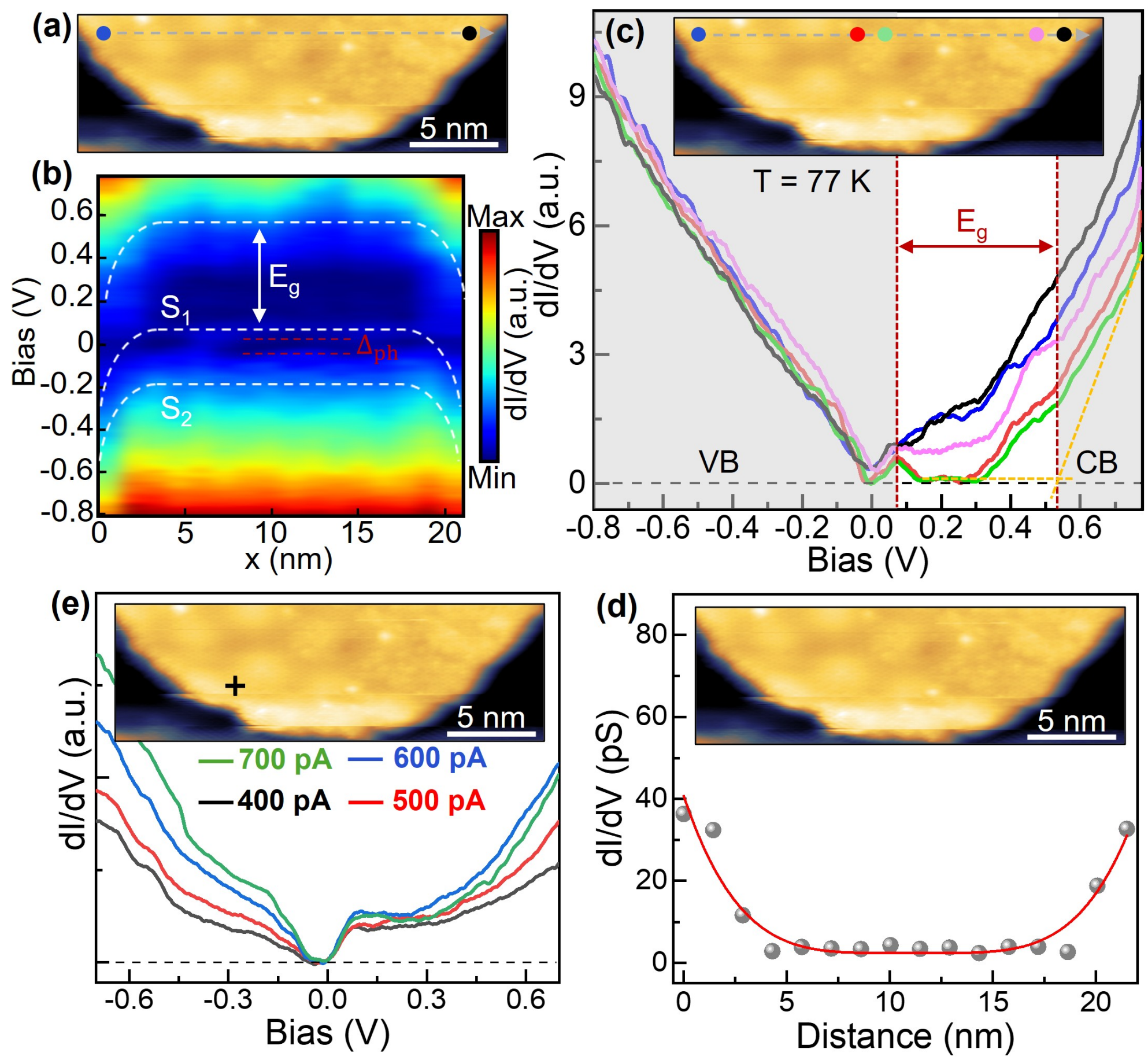


Figure S4: (a) STM topography of an intercalated bismuthene island (+1.5 V, 410 pA). (b) Line scan of $dI/dV$ spectra acquired along the gray-dashed arrow in Fig. S4a). The blue and black circles represent the start and end positions. The dashed lines indicate the energy gap of bismuthene in the bulk. Clearly visible is the modification of the band gap towards the edges. (c) Representative $dI/dV$ spectra acquired at the selected positions marked by colored circles in the inset STM image ($V_b$ = +0.8 V, $I_t$ = 400 pA). (d) Integrated $dI/dV$ signal extracted from the STS map (Fig. S4b) across the island (+0.07 to +0.3 V). The fitting curve (red) was obtained using a power-law function. (e) Set-point current-dependent STS recorded at the position (black cross) indicated in the inset image ($V_b$ = +0.8 V, $I_t$ = 400, 500, 600, 700 pA). The bulk band gap of the intercalated bismuthene layer and the phonon-induced gap are labeled $E_g$ and $\Delta_{\mathrm{ph}}$, respectively. All STM/STS data were measured at T = 77 K.

Fig. S5a) presents the $dI/dV$ spectra of the armchair edge recorded at two different temperatures. At 4.5 K, the spectrum shows an LDOS dip around $E_F$, indicating the Tomonaga–Luttinger liquid (TLL) behavior of the 1D-metallic edges. This characteristic is still observed in the spectrum (black) recorded at 77 K. The broader spectral feature (at 77 K) is attributed to thermal broadening of the tunneling effect. The edge spectrum is different from the spectroscopy of the H-intercalated ZLG (where the Dirac point is above $E_F$) and the island interior (where the bulk band gap of the underlying bismuthene contributes to the whole spectrum) (Fig. S5b). However, the phonon-induced gap of graphene prevents a quantitative analysis of the TLL behavior in the edge spectroscopy (green). The STM image in Fig. S5c) shows the topography of an intercalated bismuthene boundary, where both armchair and zigzag edges are observed. To investigate the electronic structure, the tunnelling spectra were recorded at different positions along the investigated boundary (Fig. S5d). All the spectra show a finite offset in the $dI/dV$ intensity, demonstrating the metallic nature of the bismuthene-derived edge states. Moreover, a pronounced suppression of the LDOS around $E_F$, which represents the hallmark of the TLL behavior, is observed in the spectra of both armchair and zigzag edges.[S3,S5] The edge spectral features remain unchanged at different tunneling currents (Fig. S5e). To exclude the influence of modulation voltage ($V_{\mathrm{rms}}$) on the measured STS, we also recorded $dI/dV$ spectra using two different $V_{\mathrm{rms}}$, as shown in Fig. S5g). All the STS curves exhibit similar spectral features. The robustness of edge states over a wide range of tunneling conditions and temperatures indicates that the observed zero-bias suppression is an intrinsic property of the 1D-edge channels, rather than the Efros–Shklovskii pseudogap[S6] and Coulomb blockade origins.[S7,S8]

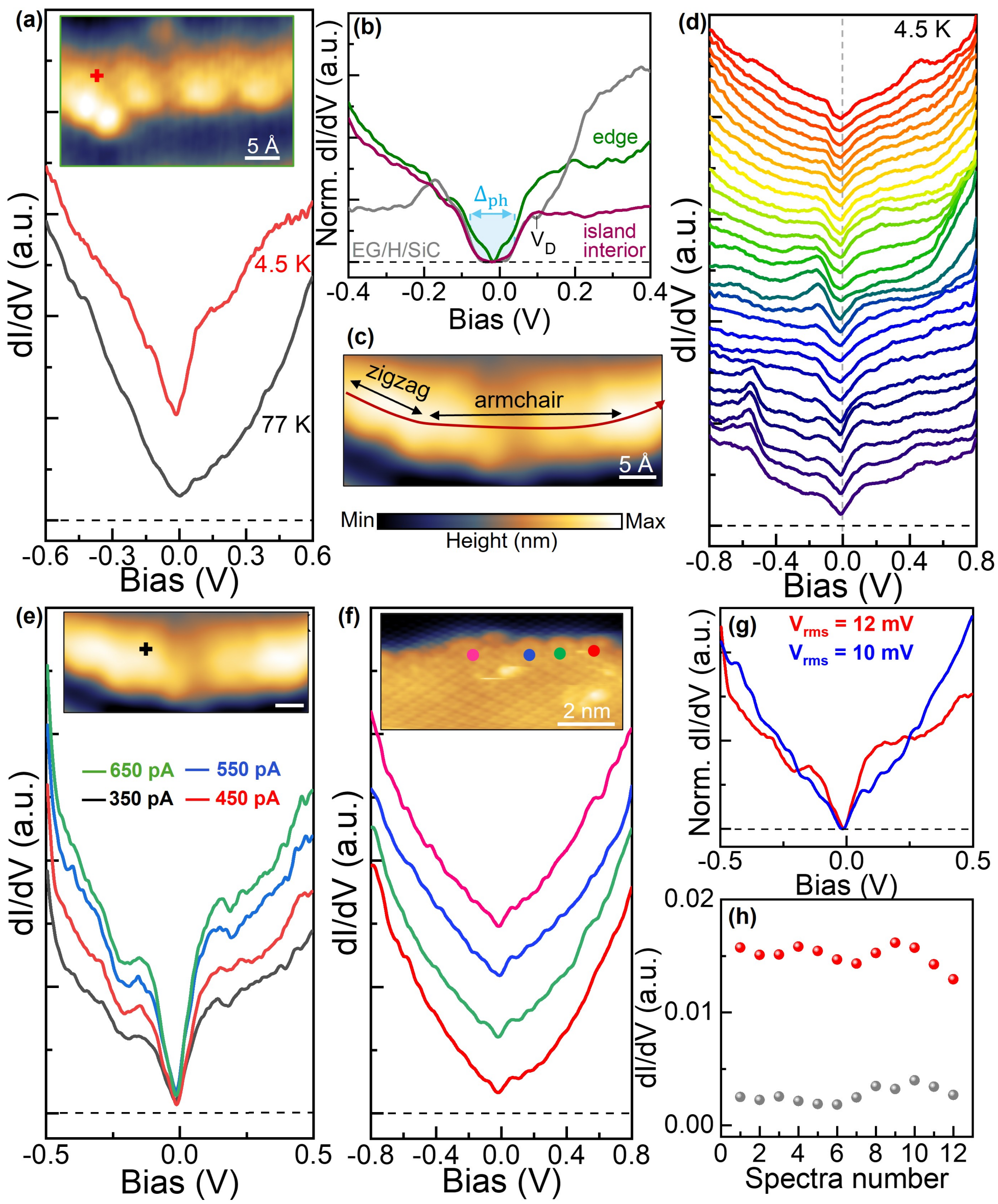


Figure S5: (a) Stacked $dI/dV$ spectra recorded at 77 K (black) and 4.5 K (red). Inset: STM image shows the measured position (red cross). (b) STS measured at an H-intercalated ZLG region (EG/H/SiC) compared to the STS recorded at an island interior and boundary. For better comparison, the spectra were normalized and shifted to the zero conductance. (c) STM topography of an intercalated bismuthene island (+0.5 V, 420 pA), showing the bismuthene armchair and zigzag edges. (d) Stacked $dI/dV$ spectra recorded at different positions along the island boundary (brown arrow). (e) Set-point current-dependent STS of the edge (black cross) recorded at $V_b = +0.8$ V. (f) STS measured at random positions (colored circles, inset STM image) along an armchair edge. (g) Comparison of the edge spectra recorded at two modulation voltages $V_{\rm rms}$. (h) Comparison of the integrated conductance (-32 mV to +32 mV) extracted from 12 $dI/dV$ spectra measured at random positions on the EG/H/SiC and the island boundary (edge). All STS data were measured at $V_b = +0.8$ V, $I_T = 450$ pA and at $V_{\rm rms} = 12$ mV (Figs. S5a-e) and $V_{\rm rms} = 10$ mV (Figs. S5f-g), T = 4.5 K. The black dashed lines represent the zero conductance.

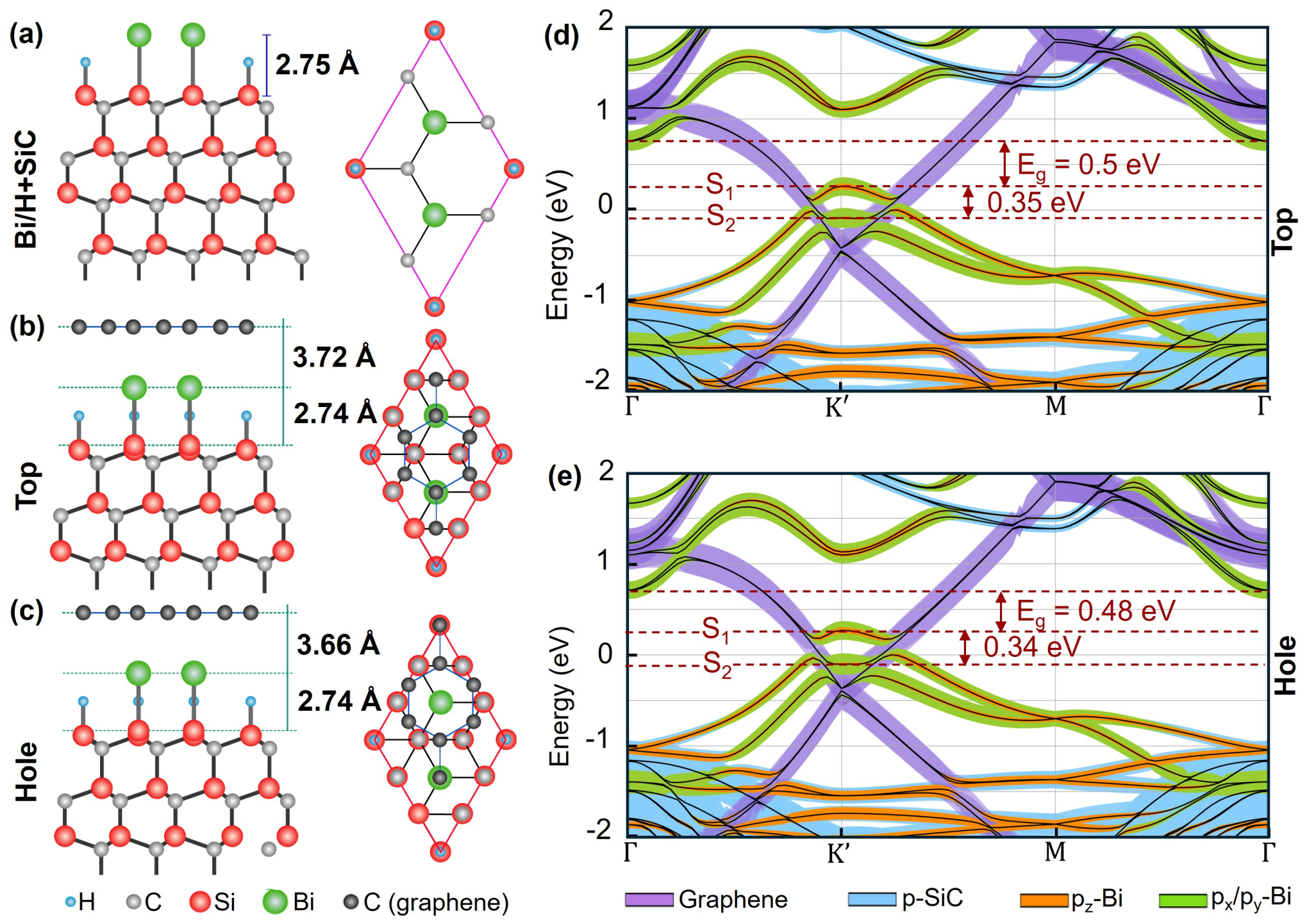


Figure S6: Atomic model displaying the interlayer distances obtained after performing DFT structural relaxations of (a) bismuthene/H+SiC, and (b-c) bismuthene-intercalated ZLG: (b) Top-site configuration and (c) Hole-site configuration. Side view (left) and Top view (right). (d-e) Corresponding orbital projected band structures of bismuthene-intercalated ZLG for the top- and hollow-site, respectively. The indirect band gap of bismuthene is denoted as $E_g$, while $\Delta_s$ represents the energy spacing between the $S_1$ and $S_2$ bands of the valence band (VB).

# Supporting information: DFT calculations

In Fig. S6a), a ball and stick model of bismuthene on SiC is presented. As shown in the figure, two of the three surface Si atoms of SiC are saturated with Bi atoms, while the remaining Si atom is passivated with hydrogen. The obtained interlayer distance between bismuthene and SiC is 2.75 Å, which is in good agreement with previous experimental reports.[S9] In Figs. S6b) and c), the optimized atomic structures for the top and hole configurations are shown, respectively. In both cases, the inclusion of the graphene layer leads to a small decrease of 0.01 Å in the bismuthene–SiC distance. The graphene–bismuthene distance differs slightly between the two configurations, being 3.72 Å for the top case and 3.66 Å for the hole case.

DFT calculations were performed for the graphene/bismuthene/SiC system. Here, an approximate structural model consisting of a $(2 \times 2)$ graphene on bismuthene/$(\sqrt{3} \times \sqrt{3})$-SiC was employed. To investigate the influence of local stacking variations on the electronic structure of the intercalated system, two distinct relative stacking configurations of graphene on bismuthene/SiC were considered (see Figs. S6b-c). As shown in Figs. S6d)–e), the graphene-derived $\pi$ bands retain their linear dispersion, with a small gap opening at the Dirac point due to orbital hybridization (top-site configuration) and sublattice symmetry breaking (hollow-site configuration). Note that in both configurations (top and hollow), due to band folding, the graphene K point coincides with the K' point of the BZ of the bismuthene layer. For the top case, where Bi atoms reside above Si atoms directly beneath graphene C atoms, the two highest-lying states $S_1$ and $S_2$ are split by $\Delta_s = 0.35$ eV owing to the Bi-derived spin–orbit coupling (SOC) and the Rashba effect.[S2,S9] The $S_1$ band lies slightly above the Fermi level because of substrate-induced p-doping, consistent with ARPES measurements.[S9] The conduction-band minimum at the $\Gamma$ point gives rise to an indirect band gap of 0.5 eV (relative to $S_1$), comparable to that of bismuthene on H-saturated SiC (0.54 eV; see main text), indicating the weak influence of graphene on the intercalated layer. A similar electronic structure is obtained for the hollow-site configuration, where Bi atoms are positioned at the centers of graphene hexagons. The indirect gap ($E_g = 0.48$ eV) and $S_1$–$S_2$ splitting ($\Delta_s = 0.34$ eV) are only marginally smaller, demonstrating that the band structure depends weakly on the adsorption site. The close quantitative agreement between both configurations confirms that the intercalated bismuthene layer is only weakly perturbed by the graphene overlayer, underscoring the robustness of the Bi-derived electronic bands against interfacial structural variations.

As shown above, Fig. S6 already indicates only minor differences between the graphene-capped bismuthene on SiC and bismuthene on SiC without the graphene layer. In Fig. S7 we compare the band structure of both cases in panel a) with graphene and panel b) without. At the K point, the graphene and bismuthene bands exhibit feature which result from

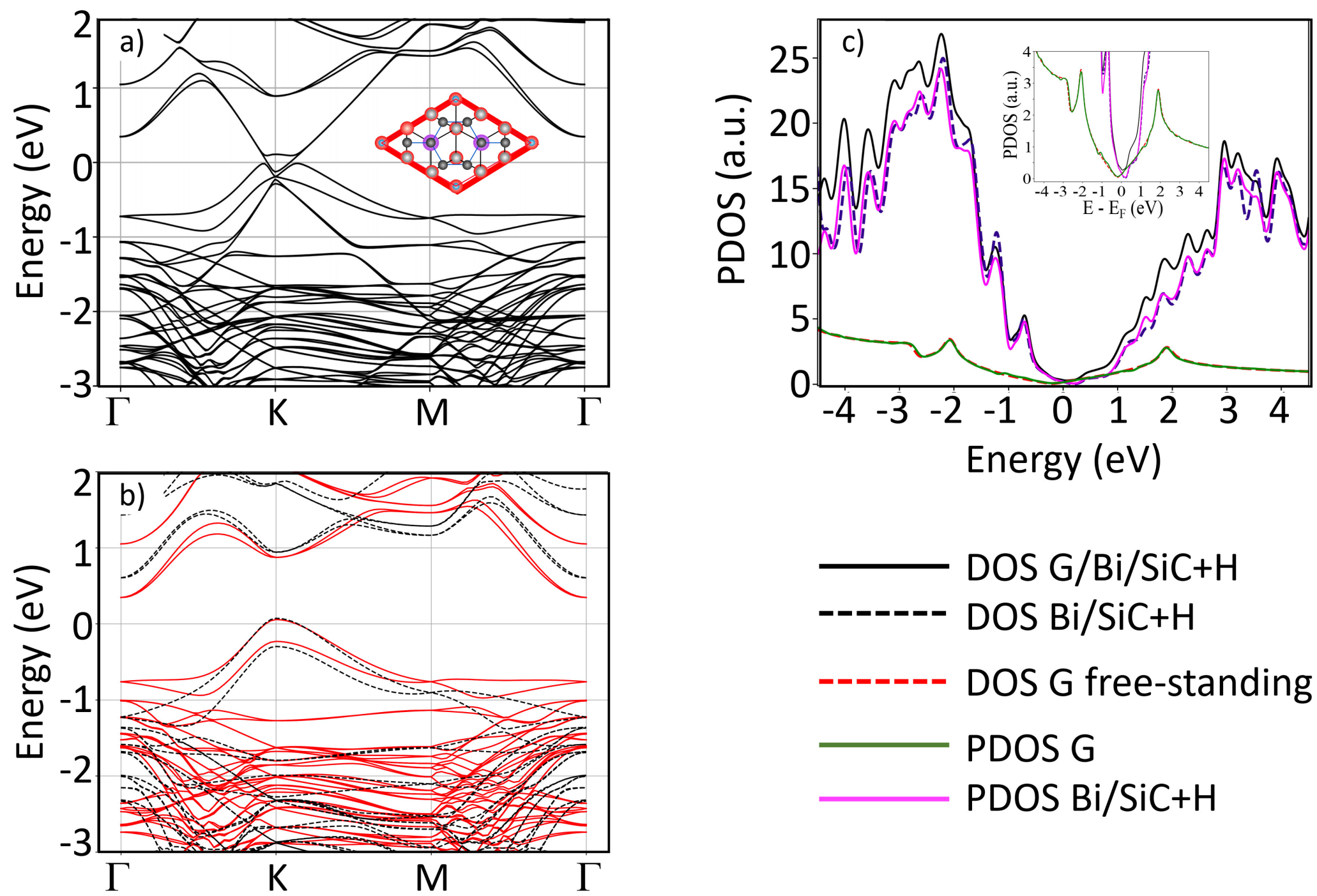


Figure S7: Comparison between the band structures of a) graphene-capped bismuthene on SiC and b) bismuthene on H-passivated SiC without the graphene layer. c) Analysis of the density of states by comparison of the layer-projected DOS curves of the system (Fig. S7a) with the separate graphene and bismuthene on SiC layers.

the symmetry reduction upon stacking both honeycomb lattices at an angle of 60 degrees. Concomitantly, the density of states (DOS) analysis in panel c) yield a difference between the DOS curves of both systems above the Fermi level. However, the projections of the DOS to the graphene layer and the bismuthene adsorbed on SiC+H reveals that the spectrally resolved electron densities in the investigated energy window (-4.5 to +4.5 eV) match identically with the DOS curves of the two separate fragments. This corroborates our conclusion that the interaction between the graphene and bismuthene layers does not involve substantial electron redistributions at the interface.

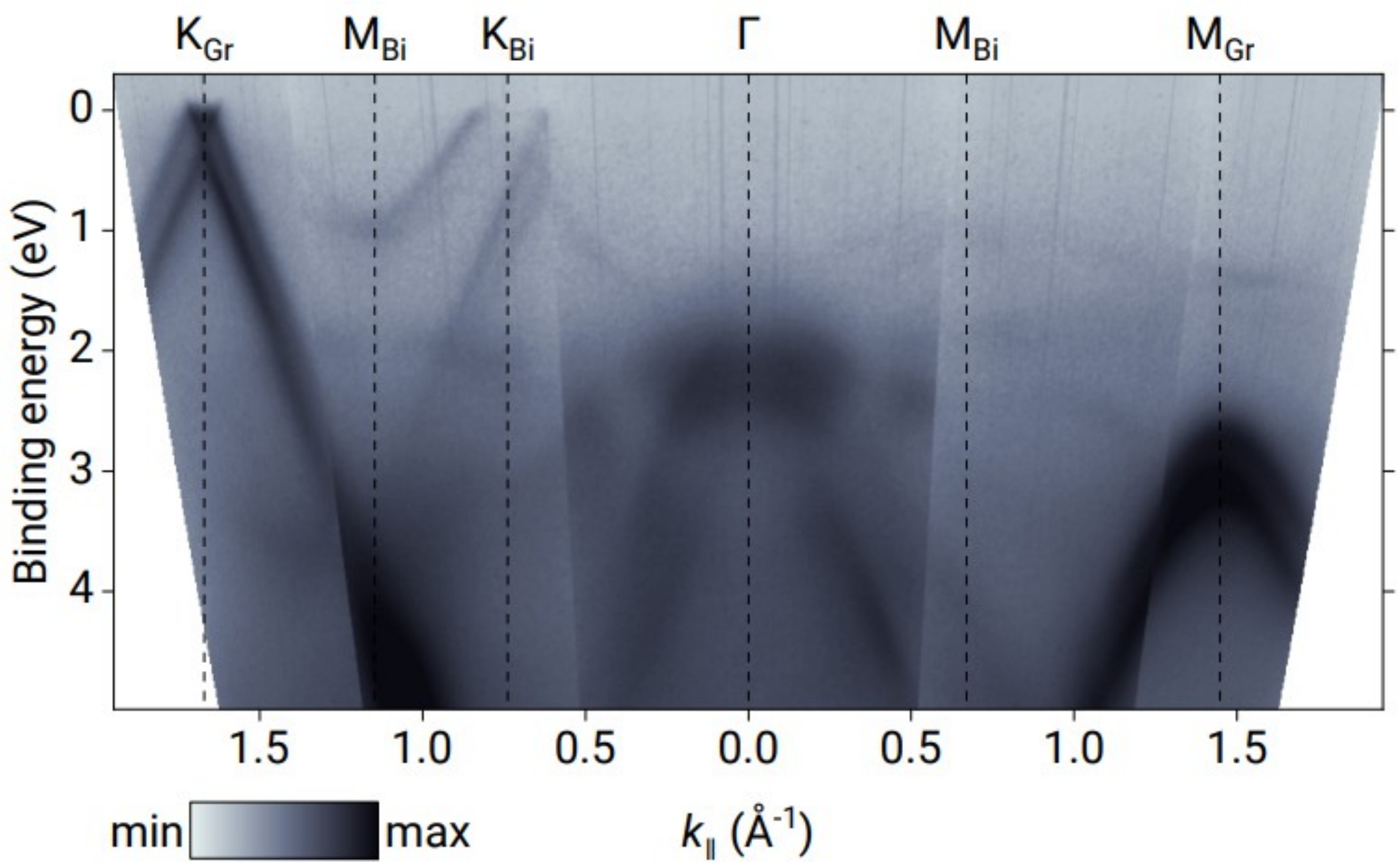


Figure S8: Energy-momentum band maps along the $\Gamma$-$K_{\mathrm{Gr}}$ and $\Gamma$-$K_{\mathrm{Gr}}$ directions of graphene, showing the electronic bands of graphene, bismuthene, and SiC.

## Supporting information: ARPES measurement

To better understand the electronic properties, we have mapped the band structure of the bismuthene intercalated ZLG sample using angle-resolved photoemission spectroscopy (ARPES), as shown in Figs. S8). The two Dirac cones with distinct carrier types are clearly visible at the graphene K point ($K_{\mathrm{Gr}}$). One originates from the graphene layer on bismuthene (EG/Bi/SiC), and the other one comes from the H-intercalated ZLG region (EG/H/SiC). In the EG/H/SiC region, the Dirac point of graphene is slightly above $E_F$, indicating that the graphene is p-doped. In contrast, the bismuthene-induced QFMLG exhibits the Dirac point below $E_F$, implying n-type doping of graphene. In addition, the bismuthene valence band - specifically the upper one - is also observed throughout the Brillouin zone (at $K_{\mathrm{Bi}}$). The Bismuthene valence band slightly shifts above $E_F$ due to the p-doping effect of the SiC substrate.[S9]

# References


(S1) Momeni Pakdehi, D.; Schädlich, P.; Nguyen, T. T. N.; Zakharov, A. A.; Wundrack, S.; Najafidehaghani, E.; Speck, F.; Pierz, K.; Seyller, T.; Tegenkamp, C.; Schumacher, H. W. Silicon Carbide Stacking-Order-Induced Doping Variation in Epitaxial Graphene. Advanced Functional Materials **2020**, 30, 2004695.

(S2) Reis, F.; Li, G.; Dudy, L.; Bauernfeind, M.; Glass, S.; Hanke, W.; Thomale, R.; Schäfer, J.; Claessen, R. Bismuthene on a SiC substrate: A candidate for a high-temperature quantum spin Hall material. Science **2017**, 357, 287–290.

(S3) Stühler, R.; Reis, F.; Müller, T.; Helbig, T.; Schwemmer, T.; Thomale, R.; Schäfer, J.; Claessen, R. Tomonaga–Luttinger liquid in the edge channels of a quantum spin Hall insulator. Nature Physics **2020**, 16, 47–51.

(S4) Zhang, Y.; Brar, V. W.; Wang, F.; Girit, C.; Yayon, Y.; Panlasigui, M.; Zettl, A.; Crommie, M. F. Giant phonon-induced conductance in scanning tunnelling spectroscopy of gate-tunable graphene. Nature Physics **2008**, 4, 627–630.

(S5) Stühler, R.; Kowalewski, A.; Reis, F.; Jungblut, D.; Dominguez, F.; Scharf, B.; Li, G.; Schäfer, J.; Hankiewicz, E. M.; Claessen, R. Effective lifting of the topological protection of quantum spin Hall edge states by edge coupling. Nature Communications **2022**, 13, 3480.

(S6) Bartosch, L.; Kopietz, P. Zero bias anomaly in the density of states of low-dimensional metals. The European Physical Journal B - Condensed Matter and Complex Systems **2002**, 28, 29–36.

(S7) Hanna, A. E.; Tinkham, M. Variation of the Coulomb staircase in a two-junction system by fractional electron charge. Phys. Rev. B **1991**, 44, 5919–5922.

(S8) Devoret, M. H.; Esteve, D.; Grabert, H.; Ingold, G.-L.; Pothier, H.; Urbina, C. Effect of the electromagnetic environment on the Coulomb blockade in ultrasmall tunnel junctions. Phys. Rev. Lett. **1990**, 64, 1824–1827.

(S9) Tilgner, N.; Wolff, S.; Soubatch, S.; Lee, T.-L.; Peña Unigarro, A. D.; Gemming, S.; Tautz, F. S.; Seyller, T.; Kumpf, C.; Göhler, F.; Schädlich, P. Reversible switching of the environment-protected quantum spin Hall insulator bismuthene at the graphene/SiC interface. Nature Communications **2025**, 16, 6171.
